# Understanding the conditions that influence the roles of midwives in Ontario, Canada’s health system: an embedded single-case study

**DOI:** 10.1186/s12913-020-5033-x

**Published:** 2020-03-12

**Authors:** Cristina A. Mattison, John N. Lavis, Eileen K. Hutton, Michelle L. Dion, Michael G. Wilson

**Affiliations:** 1 Department of Obstetrics and Gynecology, McMaster Midwifery Research Centre, 1280 Main St. West, HSC-4H26, Hamilton, ON L8S 4K1 Canada; 2 McMaster Health Forum, 1280 Main St West, MML-417, Hamilton, ON L8S 4L6 Canada; 3 Department of Political Science, McMaster University, 1280 Main St. West, KTH-533, Hamilton, ON L8S 4M4 Canada

**Keywords:** Midwifery, Case study, Political systems, Health systems, Qualitative research, Ontario, Canada

## Abstract

**Background:**

Despite the significant variability in the role and integration of midwifery across provincial and territorial health systems, there has been limited scholarly inquiry into whether, how and under what conditions midwifery has been assigned roles and integrated into Canada’s health systems.

**Methods:**

We use Yin’s (2014) embedded single-case study design, which allows for an in-depth exploration to qualitatively assess how, since the regulation of midwives in 1994, the Ontario health system has assigned roles to and integrated midwives as a service delivery option. Kingdon’s agenda setting and 3i + E theoretical frameworks are used to analyze two recent key policy directions (decision to fund freestanding midwifery-led birth centres and the Patients First primary care reform) that presented opportunities for the integration of midwives into the health system. Data were collected from key informant interviews and documents.

**Results:**

Nineteen key informant interviews were conducted, and 50 documents were reviewed in addition to field notes taken during the interviews. Our findings suggest that while midwifery was created as a self-regulated profession in 1994, health-system transformation initiatives have restricted the profession’s integration into Ontario’s health system. The policy legacies of how past decisions influence the decisions possible today have the most explanatory power to understand why midwives have had limited integration into interprofessional maternity care. The most important policy legacies to emerge from the analyses were related to payment mechanisms. In the medical model, payment mechanisms privilege physician-provided and hospital-based services, while payment mechanisms in the midwifery model have imposed unintended restrictions on the profession’s ability to practice in interprofessional environments.

**Conclusions:**

This is the first study to explain why midwives have not been fully integrated into the Ontario health system, as well as the limitations placed on their roles and scope of practice. The study also builds a theoretical understanding of the integration process of healthcare professions within health systems and how policy legacies shape service delivery options.

## Background

Although midwifery has a long tradition in Canada, the profession’s role has shifted over time. Before the twentieth century, the roles of midwives in Canada were informal, and midwives were most often women living in the community [1]. At the turn of the twentieth century, the way in which maternity care services were delivered to pregnant women changed. Preferences for physician-led and hospital-based care grew, such that by the 1920s and 1930s, midwifery existed in the ‘periphery’ of the health system and primarily in rural and remote parts of the country [2]. More recently, there has been a resurgence of midwifery, attributed at least in part to the growth of feminist ideology and what began as a social movement spread to the mainstream [3–5]. By the 1980s, a new midwifery model emerged and centred on bringing the reproductive process back into the hands of women [1]. The midwifery philosophy emphasizes an egalitarian relationship between the client and the midwife [1].

Current research evidence on midwifery care is supportive and has demonstrated that the profession delivers high-quality maternal and newborn healthcare services [6–8]. A high quality systematic review found that midwifery-led continuity models of care – compared to other models of care- were association with safe outcomes and lower rates of intervention [7]. Research on midwifery care in Canada specifically has demonstrated excellent health outcomes [9–11], and high levels of client satisfaction [12, 13].

In Canada, significant jurisdictional variability in regulation of and health system delivery arrangements for midwifery services exists. Midwifery is regulated in the majority of provinces and territories, with the exception of Yukon and Prince Edward Island [14]. New Brunswick and Newfoundland and Labrador recently regulated midwifery in 2016 and is in the process of rolling out midwifery services [15, 16]. The variability in health system delivery arrangements includes variation in terms of practice settings, the size of the workforce, integration within the health system, and percentage of births attended by midwives. For example, in 2016–2017, midwives attended 22% of the total births in British Columbia and 16% in Ontario, compared to 4% in Quebec and 3% in Saskatchewan and Nova Scotia [17].

Ontario has the largest and most established midwifery workforce in the country, with 877 practising midwives in 2017 [17]. Ontario was the first province to regulate midwifery (1994), and the profession is regulated by the College of Midwives of Ontario [18]. In Ontario, the midwifery model of care focuses on informed choice and continuity of care, and midwives provide primary care to low-risk pregnant women throughout pregnancy and labour and birth, and up to 6 weeks postpartum [19]. Choice of birthplace is also central to the midwifery model of care, with clients having the option to birth at home, in a birth centre (where available) or in a hospital setting [19]. Midwifery services are publicly funded through the Ministry of Health and Long-Term Care’s Ontario Midwifery Program [20].

Despite having the largest midwifery workforce in the country, the demand for midwifery services in Ontario is high, and, as a result, many practices have waitlists [21]. Since regulation, midwives have increasingly become recognized in Ontario as a service delivery option in low-risk maternity care services, but many women are unable to access midwifery services. These challenges are part of a broader set of issues that the province is addressing related to improving patient-centred care. Recent healthcare reforms in Ontario have focused on improving the health system by providing faster access to interprofessional care within community-based settings, marking a departure from traditional hospital-based care [22].

These broader health reforms have included midwives as primary care providers and birth centres as non-hospital settings led by midwives. The government launched two freestanding midwifery-led birth centres in 2014, which are in Ottawa (Ottawa Birth and Wellness Centre) and Toronto (Toronto Birth Centre). Although the Ottawa and Toronto birth centres are a recent initiative, the Tsi Non:we Ionnakeratstha Ona:grahsta’ Maternal and Child Centre has operated in Ontario since 1996 on the Six Nations reserve. It is staffed by Indigenous midwives who provide both traditional and contemporary midwifery care to the Six Nations community southwest of Hamilton and is funded through the province’s Aboriginal Healing and Wellness Strategy [23].

While midwifery care aligns well with the goals of broader healthcare reforms in Ontario and the province has the largest supply of midwives in the country, many continue to experience unmet needs [21]. This suggests a policy puzzle, with a gap between a government that is supportive of midwifery-led care and a health system in which the profession is relatively marginalized. Therefore, this study asks: Since the regulation of midwives (1994), in what ways and under what conditions has the Ontario health system assigned roles to the profession of midwifery as a service delivery option? These questions are answered using Kingdon’s agenda setting and 3i + E theoretical frameworks to analyze two instances of policy reform in Ontario [24, 25]. We recognize the more inclusive “childbearing person” terminology used in Canada, however, for the understanding of an international audience use the term “women” in the article [26, 27].

## Methods

### Study design

Yin’s embedded single-case study design was used to address the research question as the approach is suited to answer explanatory questions, such as the “how” and “why” (i.e., under what conditions) a particular phenomena occurs [28]. Case studies allow for an in-depth exploration, and are often used in health policy analyses when the phenomena is happening in real time and not within the researchers’ control [28]. A single case study allows the researcher to explore new theoretical relationships in order to develop a deeper understanding of the phenomena [29]. Within the embedded single-case study design, two or more embedded units of analysis are positioned within the case and context. The single-case study design was selected over a multiple-case study design, as the approach allows for more extensive analysis of the embedded units, which yields greater insights into the case [28].

### Defining and sampling the cases

**Fig. 1:**
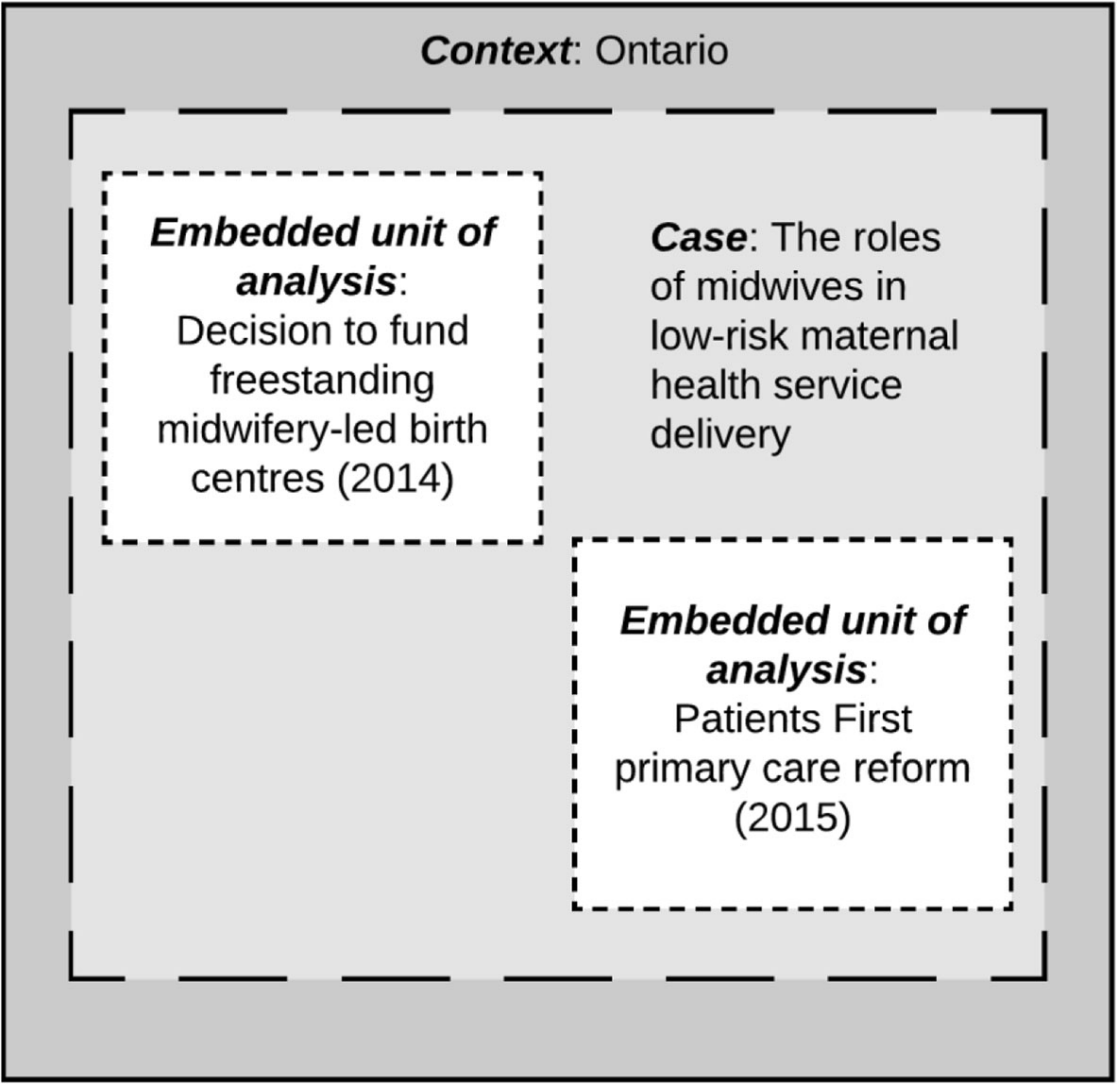
Embedded single-case study design

Figure 1 shows the embedded single-case approach used to capture the circumstances and conditions that the Ontario health system has assigned roles to the profession of midwifery as a service delivery option. The context of the study is the Ontario health system and the case is health policy-making that involves frontline maternal healthcare service providers, with a specific focus on the roles of midwives in low-risk maternal health service delivery. The two embedded units of analysis consist of recent key policy directions that presented opportunities for an increase to the roles of midwives in the health system, which are discussed below.

The first embedded unit of analysis is the decision to fund freestanding midwifery-led birth centres in 2014. Birth centres provide an opportunity and additional practice setting for midwives, yet they may also constrain the roles of midwives within the broader health system (e.g., by limiting integration of the profession into other maternity care settings). This unit of analysis explains why the Ministry of Health and Long-Term Care chose to implement birth centres in 2014, as opposed to options that could have been used to integrate midwives into acute care environments, like along-side midwifery units in hospitals.

The second embedded unit of analysis is the Patients First primary care reform, which focuses on strengthening patient-centred care [22, 30]. At the time of data collection, the Ministry of Health and Long-Term Care had released a discussion paper (*Patients First: A proposal to strengthen patient-centred health care in Ontario*) outlining the goals of the reform and soliciting input from key stakeholders including the public about implementation. The proposal was driven by four policy goals: 1) integration of services, 2) timely access to primary care services through linkages to interprofessional teams, 3) strengthening home and community care, and 4) better integration of public health [30]. While the discussion paper focused on improving patient experience through better integration and access to interprofessional primary care services, midwives were not included explicitly as part of the proposal, nor were birth centres cited as an example of community-based primary care. This unit of analysis examines why, given their scope of practice, midwives were not included as part of the Patients First primary care reform.

### Sources of evidence, sampling and recruitment

Multiple sources of evidence were collected for each embedded unit of analysis and included key informant interviews and documents (newspaper articles, published literature, policy documents, and grey literature). Data triangulation was used to develop convergent evidence for the case study [31].

A multi-stage sampling approach was used to identify and recruit key informants [32]. The first stage included identifying participants in the following five categories with experience in one or both of the units of analysis: 1) policymakers (e.g., government staff); 2) managers (e.g., managers of birth centres, midwifery regulators and members of the Better Outcomes Registry & Network Ontario); 3) healthcare providers that were involved with the policy process (e.g., midwives, primary care physicians and obstetricians); 4) consumers of midwifery services who were knowledgeable on either of the units of analysis; and 5) researchers with expertise in midwifery and/or primary care reform in Ontario. During the first stage of sampling, members of the research team identified potential participants. The second stage was driven by respondents and consisted of purposive sampling by asking research participants to identify additional key informants.

Prior to data collection, ethics approval was obtained from the Hamilton Integrated Research Ethics Board (HiREB, protocol #1266) at McMaster University in Hamilton, Ontario, Canada. Written informed consent was obtained from each participant. Invitations to participate were sent by email, with follow-up phone calls and/or emails 1 week after the initial invitation. Semi-structured interviews were either face-to-face or over the phone. The semi-structured interview guide was developed for the study and evolved over the course of interviewing to allow for clear prompts and segmentation between agenda setting (Kingdon’s framework) and policy development (3i + E framework), which is described in the subsequent section (see supplementary material for interview guide). Depending on their experience with the units of analysis, participants were asked about one or both, and the interview script was adjusted accordingly. All interviews were conducted by the principal investigator (CM) and audio recorded. The study principal investigator also took field notes during the interviews.

As is common in qualitative inquiry, analysis and interpretation overlapped with sampling and data collection. Throughout the iterative process, as transcripts and documents were analyzed, themes emerged and informed subsequent sampling and data collection. The principal investigator transcribed the audio files and transcripts were coded based on variables included in the theoretical frameworks below. The qualitative software, NVivo for Mac, was used for the organization and coding of qualitative data. Data were collected until data sufficiency was reached, when insights drawn from the analysis stages answered the research question.

The selection of the documents (newspaper articles, published literature, policy documents, and grey literature) consisted of three steps for each unit of analysis. First, a search of the LexisNexis Academic online database was used to execute the media analysis. The search string for the first unit of analysis (birth centres) included: “birth centre” AND “Ontario” in major Canadian newspapers (e.g., The Globe and Mail, The Toronto Star, and National Post). Similarly, the search string for the second unit of analysis (Patients First) included: “primary care reform” OR “patients first” AND “Ontario” in major Canadian newspapers (e.g., The Globe and Mail, The Toronto Star and National Post). Second, a search of published literature using the MEDLINE bibliographic database included the search strings “birth centre” AND “Ontario” for the first case and “primary care reform” AND “Ontario” for the second. Filters were set on publication date for articles published between 1994 (year of regulation) and May 1, 2017. Third, the grey literature search focused on policy documents, press releases, and other relevant documents. The documents were identified through Google searches using the same search strings as outlined above as well as public documents identified through the key informant interviews.

### Theoretical frameworks

**Fig. 2:**
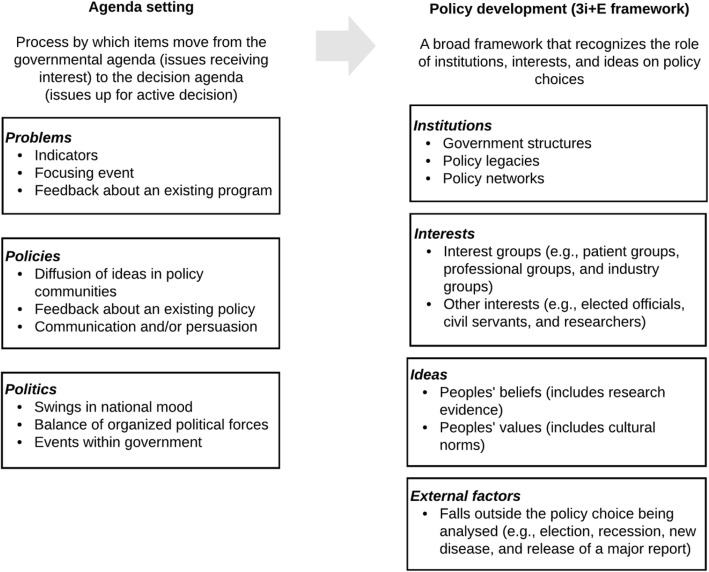
Analytic approach using Kingdon’s agenda setting and 3i + E theoretical frameworks

Figure 2 highlights the two theoretical frameworks that underpinned data analysis and focus on government agenda setting and policy development. First, Kingdon’s agenda setting framework was used to understand how the units of analysis did or did not make it to the government’s decision agenda [24]. Second, the complementary 3i + E framework was used to understand the range of factors influencing the policy choice [25]. More emphasis was placed on the 3i + E analysis as it has greater explanatory power in terms of understanding the likelihood of factors influencing policy choices.

Kingdon’s agenda setting framework recognizes the complexity of the public policymaking process and explains the process by which items move from the governmental agenda (i.e., issues that are receiving interest) to the decision agenda (i.e., issues that are up for active decision) [24]. Given all the potential topics that policymakers could pay attention to, the framework explains how and why policy issues either rise up or fall away from the agenda. The framework includes three streams: problems, policies and politics. Problems are identified as coming to attention through indicators (e.g., disease rates or rising cost of healthcare), focusing event (e.g., crisis or disaster), and/or feedback from an existing program (e.g., program may not be working as planned). Under the policy stream, possible policies to address the problem emerge from diffusion of ideas in a policy communities (i.e., progression of ideas and selection process of policy proposals), feedback about an existing policy, and communication and/or persuasion (i.e., getting the policy community to open up to a new idea). Within the politics stream, events considered as political include changes in national mood (e.g., changes in the political climate, public opinion and/or social movements), changes in organized political forces (e.g., interest group pressure campaigns) and events within government (e.g., elections and turnover in government). The governmental agenda is influenced by the problem and politics streams, while the decision agenda is influenced when the three streams come together, which is often accomplished by a policy entrepreneur who is able to influence each of the streams. In addition to the streams, the framework also considers the role of participants in agenda setting, which can be either hidden (e.g., academics, civil servants or political staff) or visible (e.g., heads of state and other politicians), as well as policy entrepreneurs.

The 3i + E framework focuses on the role of institutions, interests, ideas, and external factors on policy choices [25]. Broadly, the typology considers institutions to be government structures (e.g., federal vs. unitary government), policy legacies (e.g., how past decisions serve to influence and constrain the decisions or policies that are possible today), and policy networks (e.g., relationships between actors around a policy issue) [33, 34]. Interests can include interest groups (e.g., patient groups, professional groups, and industry groups) and other interests such as elected officials, civil servants, and researchers who may face (concentrated or diffuse) benefits and costs with particular courses of action. Ideas refer to peoples’ beliefs (including those based on research evidence) and values (e.g., cultural norms). External factors are outside of the policy choice being analysed but manifest themselves as institutions, interests and ideas (e.g., release of major reports or economic change). The 3i + E framework was applied to the two policy choices to explain how the Ontario health system assigned roles to the profession of midwifery as a service delivery option.

## Results

Thirty-seven individuals were invited to participate in the study and a total of 19 key informant interviews were completed (eight declined, nine did not reply and one could not participate due to scheduling conflicts). Participants fell into the following categories, according to their current professional role: 1) policymakers (*n* = 3); 2) managers (*n* = 3); 3) providers (*n* = 6); 4) consumers of midwifery services (*n* = 5); and 5) researchers (*n* = 2). While participants were categorized according to their current professional role, 47% (*n* = 9) of participants fell within two or more categories. The majority of the participants (*n* = 15, 79%) informed both of the units of analysis and 15% (*n* = 3) addressed exclusively the birth centre questions, while 10% (*n* = 2) focused only on the Patients First primary care reform. The interviews ranged from 25 to 68 min in length, with an average duration of 44 min. Field notes (book 1–80 pages and book 2–30 pages) were also used as sources of evidence for the interviews.

A total of 50 documents were reviewed, in addition to the key informant interviews, accompanying field notes. The following documents were included in the analysis of the decision to fund two freestanding midwifery-led birth centres: 1) newspaper articles (*n* = 10); 2) published literature (*n* = 2); 3) policy documents (*n* = 7); and 4) grey literature (*n* = 10). The following documents were included in the analysis of Patients First primary care reform: 1) newspaper articles (*n* = 3); 2) published literature (*n* = 2); 3) policy documents (*n* = 10); and 4) grey literature (*n* = 6).

We present below the findings on how and under what conditions the Ontario health system has assigned roles to the profession of midwifery as a service delivery option. We begin by presenting the results of the first embedded unit of analysis, the decision to fund freestanding midwifery-led birth centres. The section is broken down into two parts: 1) the main factors that affected government agenda setting; and 2) the main factors that influenced the likelihood of the decisions. We follow the same format to present findings related to the second embedded unit of analysis, midwifery as a service delivery option in Patients First primary care reform. Each component of the analysis is accompanied by a table which captures the core elements of the frameworks and the corresponding main themes that emerged in the analysis.

### Decision to fund freestanding midwifery-led birth centres

#### Factors that affected government agenda setting

Table 1 shows how birth centres ascended the decision agenda due to: 1) the appearance of a compelling problem; 2) a viable policy option; 3) events within the politics stream; and 4) supportive visible and hidden participants. First, attention was drawn to the problem primarily through changes in key indicators (increased rates of medical interventions in maternity care) and feedback from the operation of existing programs (e.g., restricting midwife involvement in hospital-based care). Second, the midwifery-led birth centre model rose to prominence as a viable policy option to consider through policy diffusion and from feedback from existing programs in other provinces. Third, changes in the political climate primarily influenced the politics stream through a supportive majority Liberal government. Lastly, both visible and hidden participants played an important role in moving the funding of free-standing birth centres onto the decision agenda. The then president of the Association of Ontario Midwives acted as a policy entrepreneur, taking advantage of a window of opportunity, the call for birth centre applications in 2012. When the window of opportunity opened, midwifery practice groups were able to quickly mobilize to submit applications for the Ottawa Birth and Wellness Centre and the Toronto Birth Centre.

**Table 1:** Factors that affect government agenda setting and the decision to fund freestanding midwifery-led birth centres

Factors that affect government agenda setting	Description of how these factors influenced agendas and the decision to fund freestanding midwifery-led birth centres	Sources of evidence
Problems	Rising rates of medical interventions and associated increases in healthcare costs • Increasing rates of non-medical caesareans and induction practices Hospital barriers to midwifery practice • Capping the number of midwives who have hospital privileges and the number of births attended by midwives • Restrictions to scope of practice (e.g., transfer of care criteria to an obstetrician for inductions and epidurals)	KIs [35–37]; KIs [38, 39];
Policies	Birth centre proposals and plans already existed • Original birth centre proposals and plans were available from the 1990s, which midwives were able to draw from Supportive evidence • Evidence on midwifery-led birth centre outcomes for low-risk pregnant woman in other jurisdictions in Canada (e.g., Quebec, Alberta and Manitoba)	KIs [40, 41]; KIs [42–44];
Politics	Change in government • Two original freestanding birth centres (located in St. Jacobs and Toronto) were created in the 1990s but were shelved just before doors opened due to change in government (Conservative government led by Mike Harris) when the call went out for the new birth centres, midwives were able to draw from the original applications	KIs [41];
Participants	Visible • Heavy lobbying from the then president of the Association of Ontario Midwives, which had resources to campaign (e.g., posters and blogs) • Deb Matthews, Minister of Health and Long-Term Care, was supportive of midwifery and daughter used midwives • Premier Kathleen Wynne was supportive of midwifery and used midwives for both births (in the Netherlands) Hidden • Consumers sent 10,000 electronic postcards to their MPPs, promoted birth centres on social media and at special events to promote • Midwives meeting with their local MPPs	KIs [21, 45–50]; KIs [51];

#### Factors influencing the likelihood of the decision (3i + E framework)

Within the 3i + E framework, Table 2 highlights the main factors that influenced the likelihood of the decision and policy legacies (i.e., how past decisions serve to influence and constrain the policies that are possible today) within institutions emerged as the key explanatory factor that influenced the decision to fund freestanding midwifery-led birth centres [64]. First, in a medical model, payment systems privileged physician-provided and hospital-based services, restricting the options for growth of midwifery services within primary care and hospital settings. In particular, hospital barriers to midwifery practice (e.g., capping of hospital privileges) constrained midwives, and birth centres allowed midwives to alleviate some of the pressure created by these barriers by providing an alternate practice setting. Second, the midwifery model of care limited interprofessional collaboration in hospital settings and birth centres emerged as a response to these limitations [1]. An example of limitations to interprofessional collaboration in hospital settings is that two midwives must be present at a birth and nurses cannot act as the second attendant, which has segregated midwives from other staff. Other key informants disagreed and thought that midwives holding hospital privileges facilitated interprofessional collaboration through the visibility of midwives in hospital settings.

**Table 2:** Factors influencing the likelihood of the decision to fund freestanding midwifery-led birth centres

Factors affecting policy choice	Influence on policy choice^a^	Description of how the factors influenced the decision to fund freestanding midwifery-led birth centres	Sources of evidence
Institutions	↑	**Policy legacies** Payment systems in the medical model privilege physician-provided and hospital-based services, restricting the options for growth of midwifery services • Hospital barriers to midwifery practice include: capping the number of midwives who have hospital privileges, number of births attended by midwives and restrictions to scope of practice (e.g., transfer of care criteria to an obstetrician for inductions and epidurals) • Birth centres allow midwives to circumvent barriers in hospital setting by providing an alternate practice setting	KIs [38];
	↑	Midwifery model of care is inflexible, acting as a barrier to integration and birth centres emerged as a response to these limitations (*other key informants presented an alternative interpretation, which is captured below*) • Midwives had to fight hard for regulation but as time has passed, the model (two midwives attending births) has become a barrier to integration in hospital settings as nurses cannot be seconds, which segregates midwives from other healthcare professionals • While autonomy is central to the model, it can limit interprofessional collaboration	KIs [1];
	↔	The midwifery model of care facilitates integration into the health system as midwives hold hospital privileges, which strengthens interprofessional collaboration through the visibility of midwives in hospital settings	KIs [39];
	↑	The 2008/09 increases to the number of midwifery education seats (90 total) mean that there are more new registrants looking hospital privileges and birth centres alleviate some of the pressure created by hospital barriers (e.g., capping of privileges) by offering an alternate practice setting	KIs [52];
	↓	For birth centres to be created they had to fit under existing legislation (*Independent Health Facilities Act, 1990*), as a result they are the only Independent Health Facilities that are not physician-led and birth centres are not named under the legislation or defined, which may restrict their visibility and potential for growth	KIs [53, 54];
Interests	↑	**Interest groups** • The Association for Ontario Midwives is a strong interest group and was key to lobbying for the creation of birth centres	KIs [21, 46];
Ideas	↑	**Knowledge about ‘what is’** Increasing evidence on the quality and outcomes of midwifery-led birth centres • The National Institute for Health and Care Excellence released guidelines encouraging women in the United Kingdom to give birth in midwifery-led units	KIs [55, 56];
	**↔**	Birth centres offer one possible approach to improved care for childbearing clients and there are other settings being considered by the Ministry of Health and Long-Term Care for the delivery of midwifery services • Midwifery-led care within the hospital and facilitates transfer of care when necessary (e.g., along-side birth unit in Markham-Stouffville Hospital)	KIs
	↑	**Values about ‘what ought to be’** Many women value a less medicalized approach to maternity care, as reflected by the demand for midwifery services • Many practices have wait lists for midwifery services • Not all women want to deliver at the hospital and also do not feel comfortable delivering at home, birth centres provide an alternate setting/in-between option • Many women have positive experiences midwifery care or know someone that has	KIs [21, 46, 51, 57–60];
External factors	**↔**	Professional groups finding little success within the healthcare sector have increasingly gone outside in hopes of better remuneration • In 2013 the Association of Ontario Midwives filed an application with the Human Rights Tribunal of Ontario against the Government of Ontario, citing that midwives experience a gender penalty in their remuneration (31.5%) • In early 2016 settlement talks with the Ministry of Health and Long-Term care ended without resolution and the association continues to present their case to the tribunal	KIs [61–63];

^a^Direction of arrows indicates influence on policy choice and bidirectional arrows suggest the factor neither increased nor decreased the likelihood of the policy choice

Strong interest group participation was key to lobbying efforts. Political elites, consumer campaigns and midwives meeting with local Ministers of Provincial Parliament were central to raising awareness of the birth centres initiative. The Association of Ontario Midwives was a powerful interest group that unified the profession to focus on strategic goals. For example, one interviewee shared the following regarding the role of the Association of Ontario Midwives in lobbying for birth centres:

I think the fact that we have birth centers is the result of some heavy lobbying that was done by the Association of Ontario Midwives. There have been people who have been trying to get birth centers set up for a very long time in Ontario. I think partially there is just a window there where the Association of Ontario Midwives had the resources to work hard on campaigning and there was a government that was willing to give a kick on what was a bit of a feel good option or a feel good policy that would make people feel happy. (Key informant, 27 September 2016).

Ideas influenced the decision to fund freestanding midwifery-led birth centres through values preferring a less medicalized approach to birth and supportive research evidence for midwifery-led units. Not all women wanted to deliver at the hospital, nor did they feel comfortable delivering at home, and birth centres provided an in-between setting. “Labour and birth is not an illness but it’s the point in time where a woman is at her utmost vulnerable” (Key informant, 16 September 2016).

An external factor, pay equity, also played a role in shaping the policy choice and could have added to interprofessional tensions and also further marginalized and devalued the profession within the health system [61–63]. While not directly linked to birth centres, the issue of pay equity came up in many of the key informant interviews and birth centres provide a space where midwives can practice with complete autonomy and without interprofessional tensions. Since 2013, midwives have found little success within the healthcare sector and have gone outside in hopes of better remuneration. The Association of Ontario Midwives filed an application with the Human Rights Tribunal of Ontario against the Government of Ontario, citing that midwives experience a gender penalty in their remuneration (31.5%) [61]. In early 2016, settlement talks with the Ministry of Health and Long-term Care ended without resolution, and the Association of Ontario Midwives continues to present their case to the tribunal.

### Midwifery as a service delivery option in patients first primary care reform

#### Factors that affected government agenda setting

Despite its focus on patient-centred care, midwives were not included as part of the Patients First primary care reform. The analysis presented in Table 3 explains a ‘no go’ decision (midwifery integration) within the context of a ‘go’ decision (primary care reform focused on enhancing patient-centred care). Within the problems stream, feedback from existing primary care programs, not related to maternity care, was the main factor to emerge. Policy feedback highlights that primary care reform did not take into account feedback from existing maternity care programs but rather was focused on physician-led primary care, coordination between sectors and providers, and the aging population. Changes within the organizational structure at the Ministry of Health and Long-term Care failed to effectively shape the reform agenda within the politics stream. The Ontario Midwifery Program became part of the Primary Healthcare Branch and furthered recognition of the roles of midwives as primary care providers. While the change increased the visibility of midwifery services within primary care in the ministry, it did not lead to inclusion of midwives in Patients First.

**Table 3:** Factors that affect government agenda setting in Patients First primary care reform, with a focus on midwifery as a service delivery option

Factors that affect government agenda setting	Description of how these factors influenced agendas in Patients First primary care reform, with a focus on midwifery as a service delivery option	Sources of evidence
Problems	Feedback from existing primary care programs • While most Ontarians have a primary care physician, many encounter challenges to seeing their provider in a timely manner, which leads to increased number of emergency department visits • The health system is focused acute care and reorienting the system to primary care is important to maternal health • Primary care services are at times uncoordinated, which leads to fragmentation in the system • Many experience long wait times for specialist care, which will likely increase if changes are not made to the health system due to the growth in the older adult population and those with chronic conditions	KIs [22, 30, 45, 50, 65, 66];
Policies	Supportive evidence • There has been incremental primary care reform since 2002 and feedback from existing programs, including Family Health Teams, has shown the value of team-based care • Midwives are a natural fit in primary care reform as they are primary care providers • Expanding home and community care by moving services out of hospitals and into community-based settings	KIs [67–69];
Politics	Changes within the government in terms of where midwifery is situated • The midwifery program is now part of the primary healthcare branch at the Ministry of Health and Long-Term Care	KIs
Participants	Visible • Current Minister of Health and Long-Term Care, Eric Hoskins is a midwifery consumer Hidden • Analysts at the Ministry of Health and Long-Term Care working towards reducing healthcare costs through reforming primary care • While not explicitly mentioned, the 2016 mandate letter suggests an opportunity for midwives to increase participation in primary care	KIs [70]; KIs [71];

Midwives were not integrated into Patients First primary care reform as a result of health system priorities that were focused on increasing availability and coordination of primary care services, delivered in the community by physicians and nurses. Midwives were not considered as part of the reform, most likely due to a lack of visibility within the policy arena. The lack of processes within the streams directly involving midwives, in combination with no ‘visible’ participants, acted as a constraint and dampened consideration of midwives within primary care reform.

### Factors influencing the likelihood of the decision (3i + E framework)

Within the 3i + E framework (Table 4), policy legacies also emerged as the key explanatory factors that influenced the decision not to include midwives in Patients First primary care reform. Specifically, three factors related to the policy legacies of payment mechanisms decreased the likelihood of inclusion of midwifery as a service delivery option in recent primary care reform. First, midwifery payment mechanisms limited reform by acting as a barrier to practising in interprofessional environments. One key informant summarized this point as, “because we’re funded differently, it’s made us an interloper into the primary care system.” (Key informant, 27 September 2016) Second, while not directly related to Patients First but rather broader challenges within primary care that provided context to Patients First discussions, midwifery payment mechanisms have acted as barriers to new registrants entering the workforce, as midwives can only bill for a completed course of care once the client has been discharged (typically following the 6 week postpartum visit). One key informant stated that, “there’s no other care provider in the world that cares for someone over 10 months and receives no monetary value.” (Key informant, 27 September 2016) Third, much like in the birth centre analysis, payment systems in the medical model privileged physician-provided and hospital-based services, making reform difficult and an underlying barrier to change.

**Table 4:** Factors influencing the likelihood of inclusion of midwifery as a service delivery option in Patients First primary care reform

Factors affecting policy choice	Influence on policy choice^a^	Description of how the factors influenced likelihood of inclusion of midwifery as a service delivery option in Patients First primary care reform	Sources of evidence
Institutions	**↓**	**Policy legacies** Midwifery payment mechanisms limit their ability to practice in interprofessional environments • During the 2005 primary care reforms for Family Health Teams, the call went out to midwives but they were unable to participate because they were not eligible for alternate funding arrangements	KIs
	**↓**	Midwifery payment mechanisms act as barriers to new registrants entering the workforce, as midwives can only bill for a course of care once the client has been discharged	KIs
	**↓**	Midwifery model of care is inflexible and limits the ability to be integrated into primary care teams • How midwives were regulated constrains the practice options available to them and many levers (regulatory, funding and educational) are needed to further integrate midwives into primary care teams	KIs
	↓	Payment systems in the medical model privilege physician-provided and hospital-based services, making reform difficult and an underlying barrier to change	KIs
	**↓**	Healthcare has been traditionally gendered, with priority given to physicians, which was until recently a majority male profession • Midwifery is a small workforce, providing services for women by women, which has been traditionally overlooked by the health system	KIs [1–3, 72];
	↓	Midwives are primary care providers and are a feasible option given the majority of family physicians are no longer providing maternity care due to lack flexible schedules and liability issues; however midwives are often overlooked by other regulated healthcare professionals as primary care providers in the health system	KIs
	**↔**	**Policy networks** • While tenuous at present, historically the Ontario Medical Association has had a seat at the decision-making table while the Association of Ontario Midwives has not	KIs [73, 74];
Interests	**↑**	**Interest groups** • The Association of Ontario Midwives prepared a position statement in response to the discussion paper, *Patients First: A proposal to strengthen patient-centred health care in Ontario* encouraging the Ministry of Health and Long-Term to include maternity care services in primary care reforms	KIs [38];
	↓	• Advocacy groups for older adults are larger and more established but there is a lack of formal consumer groups advocating for maternity care and midwifery services	KIs [75];
Ideas	**↑**	**Knowledge about ‘what is’** • Increasing evidence on the quality and outcomes of midwifery care	KIs [6–8, 10, 11, 76–79]; KIs [80–83];
	**↓**	• The health system is more focused on older adults and chronic disease than it is on maternity care	
	**↓**	**Values about ‘what ought to be’** • There is a disconnect between who should receive midwifery care and who seeks it (e.g., midwives provide a range of supports that are particularly important to vulnerable and marginalized populations, yet many clients are middle-class) • Social norms act as barriers and privilege physicians over midwives in maternity care • Maternity care is not on the radar like the large numbers of people dealing with caring for older adults	
External factors	**↔**	Professional groups finding little success within the healthcare sector have increasingly gone outside in hopes of better remuneration • In 2013 the Association of Ontario Midwives filed an application with the Human Rights Tribunal of Ontario against the Government of Ontario, citing that midwives experience a gender penalty in their remuneration (31.5%) • In early 2016 settlement talks with the Ministry of Health and Long-Term care ended without resolution and the association continues to present their case to the tribunal	KIs [61–63];

^a^Direction of arrows indicates influence on policy choice and bidirectional arrows suggest the factor neither increased nor decreased the likelihood of the policy choice

Midwives were less likely to be included in primary care reform due to policy legacies that prioritize physician-led care; and physicians, until recently, were more likely to be men [65]. In comparison to the primary care physician workforce, the midwifery workforce was small, providing services for women by women [1–3, 72]. While at face value midwives seemed to be a feasible option given that the majority of family physicians were no longer providing maternity care due to the lack of flexible schedules and liability issues, midwives have traditionally been overlooked by other regulated healthcare professionals as primary care providers in the health system. This is reflected in that the majority of low-risk births in the province have been attended by specialist obstetricians as opposed family physicians or midwives [38].

Interest group participation supportive of midwifery was limited, which led to a decreased recognition of the profession within Patients First. While the Association of Ontario Midwives was asked by the ministry to provide input on Patients First, it was not until after the discussion paper was released. The association submitted a position statement in response to the discussion paper, but it was one of many [38]. The submission was not prioritized given the focus of Patients First on addressing the needs of older adults and/or those with chronic conditions and the overall lack of a mobilized consumer group.

Ideational factors show that while the research evidence on quality and outcomes of midwifery care had increased, health system priorities were focused on aging, chronic disease and/or people with complex conditions (e.g., Health Links) and not maternity care [6, 7, 76]. For the first time in the country’s history, in 2016, there were more people aged over 65 than under 15 years [84]. Values were also related, in that maternity care was not on peoples’ radar like the large numbers of those who were either aging themselves or caring for older adults [80–83]. The final value that emerged was related to social norms that privilege physician-led care over midwifery-led care in the provision of maternity care services. While demands for midwifery care have increased over time, physicians and nurses still provide the majority of maternity care, and obstetricians remain the most visible maternity healthcare provider within the health system [13].

## Discussion

### Principal findings

At the time of regulation, midwives were created as an autonomous profession, yet health-system transformation initiatives have restricted further integration of midwives into Ontario’s health system. As the policy puzzle highlights, while the government has been supportive of midwifery-led care, midwives continued to have a limited role within the health system. The application of the agenda setting and 3i + E frameworks in the case study allowed for the systematic analysis of the two policy directions and identified the key factors that either helped to bolster or hinder midwifery as a service delivery option. In both cases, policy legacies held the most explanatory value. The marginalization of midwifery within primary care reform was not a result of conscious decision-making, but rather the unintended consequence of policy legacies, and show how past decisions constrain the policy options possible today [33]. The most important policy legacies to emerge from the analyses were related to payment mechanisms. In the medical model, payment mechanisms privilege physician-provided and hospital-based services, while payment mechanisms in the midwifery model have imposed unintended restrictions on the profession’s ability to practice in interprofessional environments. These findings are consistent with research on the integration of midwives into Ontario’s health system during the regulation process, which found that the health system is dominated by the medical profession and that the policy legacies contributing to this continue to influence policy processes to this day [1].

The Patients First primary care reform failed to incorporate midwives as members of the primary care team. The omission of midwives from the initiative did not emerge in the analyses as purposeful but rather a reflection that midwives and maternity care are an often-overlooked component of primary care. Health system priorities are focused on the aging population, which is a high-needs group requiring significant healthcare resources. The Canada Health Transfer has come with strings attached in terms of identifying the priority areas of home and community care and mental health and addiction services, and Patients First aligned with these strategic goals [85]. Ultimately, health system priorities were focused on responding to the perceived greater needs of the aging population and associated caregiver burden, and midwifery was overlooked in broader primary care reform.

In terms of positioning our findings within the broader literature, freestanding midwifery units in England function similarly to birth centres in Ontario. Research evidence from the Birthplace in England national prospective cohort study compared outcomes for non-obstetric unit settings (planned home births, freestanding midwifery units, and alongside midwifery units) with obstetric units [77]. The study found no significant differences in the primary outcome (composite of perinatal mortality and specific neonatal morbidities) between the non-obstetric unit and obstetric unit settings [77]. Further research on the same cohort has specifically compared freestanding midwifery units with alongside midwifery units and found freestanding midwifery units to be equally safe for babies and associated with lower rates of instrumental deliveries and higher rates of ‘straightforward vaginal birth’, which is a composite measure to describe a birth without complications that may impact subsequent pregnancies [78]. These findings are supported by a systematic review that examined maternal and perinatal outcomes in high-income countries by birth setting and found no significant differences in the odds of stillbirth or early neonatal death [86]. Subsequent research using a survey of National Health Service Trusts providing publicly funded maternity care in England mapped the provision of alongside and freestanding midwifery units, finding that there were continued challenges to the implementation of freestanding midwifery units and both in number of units and utilization [87]. Similar to our findings with birth centres in Ontario, both contexts have experienced barriers to provision of midwifery-led maternity care services [87].

### Strengths and limitations of the study

There were three mains strengths of the study. First, the embedded single-case study design allowed for the in-depth analysis of “how” and “why” midwives have been assigned roles within the Ontario health system. Second, the study design included the collection of data from multiple sources, which allowed for data triangulation. Third, the robust approach to sampling the case allowed for analysis of a policy puzzle: why would a government that has been supportive of the midwifery-led case not include the profession or birth centres in primary care reform? The embedded units of analysis were carefully selected in terms of their mapping to the study objectives. The selection of Patients First was particularly timely as ethics approval was sought within weeks of the discussion paper being released.

There was one main challenge to this study, and it related to the recruitment of key informants. Thirty-seven individuals were invited to participate in the study and 18 did not participate (eight declined, nine did not reply and one could not participate due to scheduling conflicts). Of the eight participants who declined: three declined because they did not feel they had enough experience with the cases, one was unable to participate due to a confidentiality agreement, one was sick and unable to participate, and the remaining three were from a birth centre and, while they expressed interest in participating, they ultimately did not. Participation in the key informant interviews was sought from both birth centres, and only one of the birth centres was willing to participate in the study. For the birth centre that did not participate, we were able to capture related data through participants who were healthcare providers holding privileges and practising at the site.

## Conclusions

As primary care reform continues in the province, we hope the study will be useful to policymakers and healthcare providers in understanding the key policy legacies that influenced policy directions. Despite a government that is supportive of midwifery services, they are often overlooked in policy decisions. The research findings suggest that midwives need an institutional voice in primary care policy conversations. Specifically, meetings related to primary care policies that have representation from physicians and nurses should ideally not occur without midwives at the table. Identifying critical junctures, moments when substantial institutional change takes place thereby creating a ‘branching point’ from which historical development moves onto a new path, are key to moving midwifery forward and past the constraints created by policy legacies [34].

The case study is timely, as there is jurisdictional variability across provinces and territories in Canada, with midwifery remaining unregulated in a few jurisdictions. Ontario has emerged as a leader in midwifery as it was the first province to regulate the profession, has the largest and most established workforce, and trains the most midwives in the country. Supportive evaluations from the first year of operations of the two birth centres have found that clients had significantly fewer interventions and care reflected current best practices [88]. The findings from the evaluation are consistent with research internationally and support the safety of midwife-led out-of-hospital births for low-risk populations [77–79, 89]. Understanding the conditions under which midwifery has been assigned roles within the Ontario health system is important not only to this particular policy puzzle but has implications for international contexts and health systems in Canada making policy decisions regarding the integration of midwifery services.

## Supplementary information

**Additional file 1.** Interview guide. Copy of the semi-structured interview guide that was developed for the study to guide the key informant interviews.

## Data Availability

All data contributing to the analyses in this study are stored on a secure network and not publicly available in order to protect participant confidentiality.
