# Supplementary material for: Understanding the conditions that influence the roles of midwives in Ontario, Canada’s health system: an embedded single-case study
Source: BMC Health Serv Res. 2020 Mar 12;20:197. doi: 10.1186/s12913-020-5033-x (PMC7068956; doi:10.1186/s12913-020-5033-x)
Supplement: Supplementary file 1 — Additional file 1. Interview guide. Copy of the semi-structured interview guide that was developed for the study to guide the key informant interviews. [file 12913_2020_5033_MOESM1_ESM.docx]

# Interview guide

Understanding the role and integration of midwifery in Ontario's health system

**Ethical considerations:**

A description of the study will have been presented during the recruitment phase. A signed confirmation of commitment to participate will be obtained prior to engaging in the questions. Any ethical issues arising will be addressed prior to the first question and will be documented by the Interviewer.

**Process:**

Interviews will be recorded on a digital audio device, transcribed, and uploaded into a qualitative software program. Handwritten notes will also be made by the interviewer into her field notebook.

- Denotes probes

| Date: |  |
| --- | --- |
| Time: |  |
| Place: |  |
| Interviewer: |  |
| Interviewee: |  |
| Position of Interviewee: |  |

**Questions**

1. Do you have any questions for me before proceeding to the interview?
2. Could you describe, in general, your experience related to the [policy focus: 1) creation of two midwifery-led birth centres in 2014, and/or policy issue 2: recent primary care reform discussion paper (*Patients first: a proposal to strengthen patient-centred health care in Ontario)*].
3. Based on your understanding of the issue, what do you think the policy [proposal] was designed to address?
4. How did the issue come to the attention of stakeholders and policymakers?
   1. Why do you think the issue was placed on the agenda?
5. How did it become a priority issue?
   - Probe for policy development and specific insights into institutions, interest groups, ideas, and external factors (3i+E framework)

Question specific to midwifery-led birth centres

1. Birth centres offer a community-based setting for the delivery of midwifery services. What other options were considered as part of the reform (e.g., midwives in acute care environments)?

- Does this approach strengthen the role of midwives into the health system?
- Why this approach, given all the other options?

Question specific to primary care reform:

1. The Patients First (2015) discussion paper focuses on improving patient experience through better integration and access to interdisciplinary primary care services (e.g., community-based care). What healthcare professions are being considered as a part of this reform?
2. What collaborative care models are being considered as a part of this reform?

- Are midwives considered as part of this reform?
- Are birth centres considered as part of this reform?
- Why or why not?

1. What are the goals of the policy(ies)? How will they be achieved/Have they been achieved?

- Improving patient experience
- Efficiency and cost-effectiveness
- Improving access to community-based care
